# Genetic Diversity in Insect Metal Tolerance

**DOI:** 10.3389/fgene.2017.00172

**Published:** 2017-11-07

**Authors:** Thomas J. S. Merritt, Adam J. Bewick

**Affiliations:** ^1^Department of Chemistry and Biochemistry, Laurentian University, Sudbury, ON, Canada; ^2^Department of Genetics, University of Georgia, Athens, GA, United States

**Keywords:** insect metal response, metallothionein, antioxidant metabolic enzymes, epigenetic regulation, ABC transporter genes

## Abstract

Insects encounter a variety of metals in their environment, many of which are required at some concentration for normal organismal homeostasis, but essentially all of which are toxic at higher concentrations. Insects have evolved a variety of genetic, and likely epigenetic, mechanisms to deal with metal stress. A recurring theme in all these systems is complexity and diversity; even simple, single gene, cases are complex. Of the known gene families, the metallothioneins are perhaps the best understood and provide good examples of how diverse metal response is. Interestingly, there is considerable diversity across taxa in these metal-responsive systems, including duplications to form small gene families and complex expression of single loci. Strikingly, different species have evolved different mechanisms to cope with the same, or similar, stress suggesting both independent derivation of, and plasticity in, the pathways involved. It is likely that some metal-response systems evolved early in evolutionary time and have been conserved, while others have diverged, and still others evolved more recently and convergently. In addition to conventional genetics, insects likely respond to environmental metal through a variety of epigenetic systems, but direct tests are lacking. Ultimately, it is likely that classical genetic and epigenetic factors interact in regulating insect metal responses. In light of this diversity across species, future studies including a broad-based examination of gene expression in non-model species in complex environments will likely uncover additional genes and genetic and epigenetic mechanisms.

## Introduction

Insects respond to environmental metals in multiple ways; here we highlight the genetics of this response and focus on the diversity of responses including different genes, gene families, and epigenetic responses.

### Metal Biology

Metals present a biological challenge in that many are absolutely required for various cellular functions, but higher concentrations are generally toxic (Southon et al., 2013). Organisms must carefully balance metal uptake, exclusion, and excretion to ensure that metals are present in sufficient, but not toxic, concentrations. Biological metal concentrations are, however, low and environmental exposure issues are generally issues of contamination leading to excess metals and toxicity. The genetics of metal response and detoxification is strikingly dynamic with independent evolution of different mechanisms to achieve this balance across animals and even specifically within insects.

Metals have a suite of biochemical functions and metal responses are similarly diverse. Many metals, e.g., iron (Fe), copper (Cu), chromium (Cr), manganese (Mn), and zinc (Zn) have key biological roles including functions in energy production, carbohydrate and lipid metabolism, and gene regulation (Bondy, 2016). Excesses of these metals, however, have both direct and indirect toxic effects (Valko et al., 2005; Southon et al., 2013). High concentrations of metals bind to DNA causing direct damage or disrupting gene expression. Heavy metals can displace essential metals and, for example, disrupt enzyme function. Such disruption can lead to oxidative stress (OS) and a suite of changes in gene expression. Metal toxicity can also result from OS caused by redox cycling metals, e.g., Fe and Cu, directly promoting generation of reactive oxygen species (ROS) and reactive nitrogen species (RNS; Valko et al., 2005, 2015). Similarly, even metals that are redox inert can result in OS toxicity by depleting antioxidants such as glutathione or by direct binding to proteins through sulfhydryl groups (Valko et al., 2015).

### Gene Systems

Dynamism is a defining characteristic of the genetics of metal response with a diversity of genes and mechanisms present across taxa. Some systems appear to be evolutionarily ancient while others are more recent adaptations and can be variable even within populations. Laboratory, studies of the physiological responses to exposure to high-metal concentrations have identified a series of metal-responsive genes. These genes function to maintain metal balance by sequestering metals and promoting their expulsion from the organism and are up regulated in response to high metal concentrations. Changes in gene expression can be functions of binding of regulatory factors, changes in chromatin accessibility or transcript stability (Janssens et al., 2009). The changes in gene expression either result directly from metal binding or indirectly through changes in redox state (Valko et al., 2005, 2015). In addition, protein concentration and activity can be modified by control of translation or post-translational regulation or stability (Janssens et al., 2009).

Metal-responsive genes have also been identified through evolutionary studies of adaptation to high metal environments. Metal tolerance has evolved in a variety of insects with interesting differences that likely reflect differences in life history and ecology. Environmental metals are often highly persistent, placing long-term selective pressures on local populations (Stone et al., 2001; Migula et al., 2004). Lacking the ability to relocate, low dispersal organisms can be continuously exposed to metals for many generations placing a high selective pressure on adaptation through genetic mechanisms, with stress-resistant members of a population more reproductively successful in contaminated sites (Migula et al., 2004). Conversely, high dispersal organisms may not develop metal tolerance as dispersal can prevent local adaptation. Evolutionary studies explore standing genetic diversity in populations and complement physiological studies, generally identifying similar genes or pathways, but with the potential to uncover novel systems especially as more diverse taxa and environments are incorporated into studies. Such an evolutionary approach identified a broad, transcriptome-wide, metal response in springtails, *Orchesella cincta*, invertebrate hexapods, from contaminated mine sites (Roelofs et al., 2009). Individuals from control and contaminated sites were exposed to cadmium (Cd) in the laboratory and the reference population, but not the tolerant population, showed a strong transcriptome-wide stress response. Further, some stress-responsive genes were less inducible in tolerant springtails, suggesting that these genes were already overexpressed in this population. These differences in expression are likely evolutionary adaptation of the population from the contaminated site; changes in gene regulation that make a major contribution to the evolution of a stress-adapted phenotype.

Fundamentally, organisms have limited energy supplies and there are costs to living in stressful environments, even “tolerant” organisms must expend metabolic resources in accommodating stressors (e.g., the metabolic cost of sequestering or expelling excess metals; Stone et al., 2001; Morgan et al., 2007). Resistant individuals are better able to handle this stress, but resistance likely comes at a cost. For example, in the absence of metal contamination, metal tolerant mosquitoes have significantly lower viability, survivorship, and fecundity than controls; their adaptations to high metal are physiological burdens in the absence of metals (Mireji et al., 2010b). In addition, metal stress can limit an organism’s ability to cope with other stressors and studies of multiple stressors have led to discoveries of novel mechanisms. For example, while carabid beetles living in a pollution gradient with high levels of Zn, Cd, Cu, and lead (Pb) were phenotypically indistinguishable from controls, they were less tolerant to desiccation or pesticide exposure (Stone et al., 2001). Multiple stressors had a profound effect; the stressed population was more susceptible to additional stressors, but it was unclear if this was a physiological effect or a function of reduction in genetic variability in the population as a result of long-term contamination. This cost of resistance may, in part, account for the dynamic nature of many genetic mechanisms of metal tolerance.

In addition to highlighting the compounding effects of multiple stressors, evolutionary studies also indicate that responses to different metal stressors may be distinct and complicated, with limited overlap. A laboratory study of. *magna* (Crustacea) exposed to Cu, Cd, or Zn, found a broad suite of genetic changes, but only four genes that were differentially expressed under all three metals; the different metal stressors resulted in distinct responses (Poynton et al., 2007). A similar study of *Fundulus heteroclitus* (Actinopterygii) exposed to a suite of contaminants showed broad changes in gene expression in different populations with only limited convergence: only two genes changed expression in all three comparisons and only one of these changed in the same direction (Fisher and Oleksiak, 2007). Limited parallel responses to metal stressors are observed between insects. Initial studies of the evolution of insecticide resistance, a stressor similar to metal contamination, in *Drosophila melanogaster* (Daborn et al., 2002; Le Goff et al., 2003), suggested that resistance was a single-gene phenomenon. However, later studies indicate that multiple genes are likely involved (Pedra et al., 2004; Schmidt et al., 2010; van Straalen et al., 2011). Additionally, populations do not necessarily adapt to a high metal environment. For example, 10 generations of high exposure to Cd or Zn led to only a moderate increase in Cd tolerance, no Zn tolerance, and no evidence for cross-resistance in *Spodoptera exigua* moths (Kafel et al., 2014). Furthermore, moths from high Zn exposure were less tolerant to Zn, possibly reflecting long-term physiological stress and an inability to adapt to the stressor.

Substantial genetic diversity in environmental metal response, within and between, species is a recurring theme in these studies. Different species respond differently to the same stressors and different stressors elicit different responses within a single species and across species. Metal tolerance is rarely, if ever, a single gene affair, and a network view is most appropriate (van Straalen et al., 2011). This point notwithstanding, a few key genes and gene families have repeatedly been found to be involved in metal tolerance (**Table 1**).

**Table 1:** Metal responsive genes in insects.

Gene family	Species	Known interacting metals
Metallothioneins	*Drosophila melanogaster*^1,2^, *Anopholese gambiae*^3,4^, *Orcista cincta*^5^	Cu, Cd, Zn
Cytosolic superoxide dismutase	*Pterostichus oblongopunctatus, Geotrupes stercorosus, Staphylinus caesareus, Phyllobius betulae*^6^	Pb, Zn, Cu, Cd
Mitochondrial superoxide dismutase	*Drosophila melanogaster*^7^	Al, Fe
Catalase	*Pterostichus oblongopunctatus, Geotrupes stercorosus, Staphylinus caesareus, Phyllobius betulae*^6^*, Drosophila melanogaster*^7^	Pb, Zn, Cu, Cd
Glutathione-*S*-transferase	*Pterostichus oblongopunctatus, Geotrupes stercorosus, Staphylinus caesareus, Phyllobius betulae*^6^	Pb, Zn, Cu, Cd
Glutathione reductase	*Pterostichus oblongopunctatus, Geotrupes stercorosus, Staphylinus caesareus, Phyllobius betulae*^6^	Pb, Zn, Cu, Cd
Se-dependent glutathione peroxidase	*Pterostichus oblongopunctatus, Geotrupes stercorosus, Staphylinus caesareus, Phyllobius betulae*^6^	Pb, Zn, Cu, Cd
Se-independent glutathione peroxidase	*Pterostichus oblongopunctatus, Geotrupes stercorosus, Staphylinus caesareus, Phyllobius betulae*^6^	Pb, Zn, Cu, Cd
ABC transporter proteins	*Drosophila melanogaster*^8^*, Lygus hesperus*^9^	Cd
α-tubulin	*Anopholese gambiae*^3^, *Chironomus tentans*^10^	Cu, Pb, Cd
CYP6	*Anopholese gambiae*^11^	Cd, Pb

*Sources: ^1^Egli et al., 2006; ^2^Atanesyan et al., 2011; ^3^Shaw et al., 2007; ^4^Mireji et al., 2010a; ^5^van Straalen et al., 2011; ^6^Migula et al., 2004; ^7^Wu et al., 2012; ^8^Sooksa-Nguan et al., 2009; ^9^Hull et al., 2014; ^10^Mattingly et al., 2001; ^11^Musasia et al., 2013*.

## Classical Genetic Responses

### Metallothioneins (MTs)

Metallothioneins (MT), small, cysteine-rich, proteins that bind metals including Cu, Zn, and Cd through metal-thiolate bridges, are one of the more thoroughly studied metal-responsive gene families (Kägi, 1991). MT gene expression is up-regulated by metals in a variety of taxa and MT gene expression has been developed as a biomarker of metal contamination in insects and other animals (Mireji et al., 2010a; M’kandawire et al., 2017). MTs are ubiquitous in eukaryotes, generally present as multi-gene families; e.g., *D. melanogaster* have at least five (Atanesyan et al., 2011) and mosquitoes (*Anopheles gambiae*) at least two (Shaw et al., 2007). These gene families likely allow functional specialization. Specific loci are differentially regulated, at both transcription and translation (Janssens et al., 2009; Mireji et al., 2010a), with specific loci expressed in response to different stimuli in specific tissues and developmental stages (Atanesyan et al., 2011; Baurand et al., 2015; Qiang et al., 2017). Different MT proteins also have distinct biochemistry, at least in *Escherichia coli* expression systems (Achard-Joris et al., 2007). Interestingly, diversity within these gene families appears to have evolved independently in different taxonomic groups, through independent evolutionary radiations and losses, not an early diversification (Guirola et al., 2010). Even within insects and other invertebrates, the MT gene family is highly diverse likely the result of independent evolutionary events and convergence on a shared pattern of diversity (Shaw et al., 2007; Janssens et al., 2009).

Metallothioneins are well studied in *D. melanogaster* (e.g., Egli et al., 2006; Guirola et al., 2010; Atanesyan et al., 2011). These flies have five known *Mt* loci, *MtnA*, *MtnB*, *MtnC*, *MtnD*, *MtnE* (Atanesyan et al., 2011; Qiang et al., 2017). These loci are all induced by a shared transcription factor and shared promoter response element, but each has distinct expression patterns and likely distinct, but possibly overlapping, functions (Egli et al., 2006; Atanesyan et al., 2011; Qiang et al., 2017). Interestingly, given the diversity of this gene family across taxa, the five loci, and their expression products are conserved across 12 species of Drosophila (Guirola et al., 2010); the family can remain stable across considerable evolutionary time. The *D. melanogaster* MT loci are all primarily expressed in the intestine, but the different loci appear to respond to different metals, e.g., *MtnA* is preferentially induced by Cu and *MtnB* by Cd (Atanesyan et al., 2011; Qiang et al., 2017). The variable gene expression is reflected in different amounts of protein and the proteins have distinct biochemical characteristics, e.g., distinct binding affinities for Zn, Cd, and Cu (Egli et al., 2006; Qiang et al., 2017). *MtnE*, is the most recent *D. melanogaster* locus to be described (Atanesyan et al., 2011), has the broadest metal binding affinity, and may be a general-metal response element (Pérez-Rafael et al., 2012). *MtnB* gene expression, and *MtnB* mutant flies, were sensitive to Cu, Cd, and Zn. *MtnB*, *MtnC*, and *MtnD* expression also respond to elevated Fe levels (Qiang et al., 2017), but this response is indirect, a result of interruption of protein interactions with other metals, such as Zn, highlighting that metal toxicity can result from more complicated interactions than simple direct metal binding or displacement, adding an additional level of complexity to the genetics of metal response (Qiang et al., 2017). Further, expression of MT proteins has also been associated with induction, rather than repression, of OS, possibly a function of release under oxidizing physiological conditions of MT-bound metals that then interact with the superoxide dismutase (SOD) system (Achard-Joris et al., 2007). This counterintuitive association of a metal-tolerance gene family with induction of OS further highlights the complexity of these stress response elements.

Metallothioneins have also been studied in other insects and invertebrates. Expression of at least one of the two mosquito MT loci responds to Cd and Pb (Mireji et al., 2010a). Interestingly, in contrast to the multiple loci in fruit flies and mosquitoes, the springtail invertebrate hexapod, *Orchesella cincta*, has a single MT locus. Expression of this single locus is metal-responsive and expression differences appear to reflect local adaptation to metal contamination (Janssens et al., 2009; Roelofs et al., 2009; van Straalen et al., 2011). Further, regulatory variation in the *O. cincta* MT gene is sensitive to genetic background, i.e., an allele behaves differently in different backgrounds, indicating that expression is modified by *trans*-acting factors (van Straalen et al., 2011) and highlighting that even this “simple” system is complex, the function of multiple genes.

### Antioxidant Metabolic Enzymes

Metal exposure is associated with OS and antioxidant enzymes have a substantial role in insect metal response. Metal exposure variously results in either enhancement or inhibition of antioxidant enzymes including mitochondrial and cytosolic SOD, catalase (CAT), and Glutathione-*S*-transferase (GST; Migula et al., 2004). Aluminum (Al) toxicity in *D. melanogaster* is a function of interactions with Fe, is mediated by ROS production, and results in increased expression of mitochondrial SOD, but not cytosolic SOD and is suppressed by overexpression of CAT, but not SOD (Wu et al., 2012) again highlighting the potential for indirect interactions and the complexity of genetic responses. Beetles exposed to a complex mix of pollutants including high levels of Pb, Zn, Cd, and Cu, had significant changes in the activities of antioxidant enzymes (Migula et al., 2004), but different species responded differently, possibly reflecting differences in life history leading to different effective exposures, or differences in genetic response in each species, or both. While exact responses differed across species, in general SOD activity was high, and CAT activity low, in highly contaminated areas and activity of glutathione enzymes were more variable. Strikingly, all enzyme activities were most variable in highly contaminated environments; stressful environments expose biological variability (Migula et al., 2004). This is exactly the kind of variability that would be required for evolutionary adaptation of these stressful environments (van Straalen et al., 2011).

### Additional Gene Families

A wide variety of other genes and gene families have been directly or indirectly implicated in insect metal response (Calap-Quintana et al., 2017). The ATP-binding cassette (ABC) transporter proteins, a large family of membrane proteins characterized by an ATP-binding cassette, have a variety of biological roles, including metal detoxification. Metal-responsive ABC transporter genes are present in insects (Sooksa-Nguan et al., 2009; Hull et al., 2014) and, in *D. melanogaster*, increased gene expression is associated with tolerance to metal exposure (Sooksa-Nguan et al., 2009). Cadmium and Pb tolerant *A. gambiae* mosquitoes have significant reduction in expression of the CYP6 family of cytochrome p450 genes, a gene family associated with resistance to pyrethroid pesticides (Musasia et al., 2013), suggesting potential interactions between metal tolerance and pesticide sensitivity. Expression of the alpha-tubulin gene also responds to metal concentrations in both *A. gambiae* (Mireji et al., 2010a) and *Chironomus tentans* midges (Mattingly et al., 2001). This is not an exhaustive list, and as more efforts are put in to broad-based exploratory studies (see below) it will continue to grow.

## Epigenetic Systems

Insect metal response likely extends beyond classical genetics to epigenetics – inherited phenotypic change that is not solely due to a change in DNA sequence. Epigenetic changes appear to contribute to metal tolerance in both animals (Salnikow and Zhitkovich, 2008) and plants (Hanikenne and Nouet, 2011), but have yet to be functionally tested in insects. DNA methylation to the 5th atom of cytosine (5-methylcytosine; 5 mC) is a relatively well-studied epigenetic modification in other organisms, and an attractive modification to test for an epigenetic component to metal stress in insects. In mouse cells, for example, 5 mC variation at the promoter of a MT gene causes differences in expression; 5 mC changes histone associations and represses gene expression (Majumber et al., 2006). Additionally, in the plant *Arabidopsis thaliana*, 5 mC variation is associated with variation for biotrophic bacterial pathogen resistance (Reinders et al., 2009). However, 5 mC is absent from *D. melanogaster*, and Diptera universally (Raddatz et al., 2013; Bewick et al., 2016), hindering functional testing and leading to speculation that epigenetic regulation by 5 mC has a smaller role in insects. Species belonging to other orders of insects do, however, possess varying levels and genomic locations of 5 mC (Kharchenko et al., 2011; Bewick et al., 2016; Glastad et al., 2016a,b, 2017). The functional importance of this variation specifically in response to metals, and stress generally, has yet to be tested. However, tractable species for epigenetic functional studies have been recently identified. An insect epigenetic response to metal stress will likely be complex, but systems and tools are now at hand to move insect functional epigenetics forward (Trible et al., 2017).

## Future Directions

Complexity of genes, gene families and regulatory mechanisms is a recurring theme in insect metal response. Our current understanding strongly suggests that even more complex systems exist and calls for broad-based exploration across species taking advantage of recent technical advantages in transcriptomics, proteomics, and metabolomics (Morgan et al., 2007; Poynton et al., 2007; Shaw et al., 2007; Soetaert et al., 2007). The last two fields are particularly understudied although recent work indicates that moving beyond the transcriptome can uncover unexpected, but biologically important, interactions (e.g., Knee et al., 2013; MacMillan et al., 2016).

The evolution of different metal-tolerance mechanisms also suggests that we need to explore both with established model systems and novel systems. Recent advances in gene editing technologies should open up such novel systems for study (Janssens et al., 2009). In addition, existing work indicates that multiple stressors result in different responses than single stressors and genetic differences between individuals may only be apparent under a complex set of environmental conditions (Whitehead and Crawford, 2006; Alvarez et al., 2015). Studies of novel systems in the wild or other complex stress conditions is likely, then, to identify novel resistance mechanisms (Colbourne et al., 2011; Alvarez et al., 2015).

## Author Contributions

All authors listed have made a substantial, direct and intellectual contribution to the work, and approved it for publication.

## Conflict of Interest Statement

The authors declare that the research was conducted in the absence of any commercial or financial relationships that could be construed as a potential conflict of interest.
